# Genomic Characterization of Jumbo *Salmonella* Phages That Effectively Target United Kingdom Pig-Associated *Salmonella* Serotypes

**DOI:** 10.3389/fmicb.2019.01491

**Published:** 2019-07-02

**Authors:** Anisha M. Thanki, Nathan Brown, Andrew D. Millard, Martha R. J. Clokie

**Affiliations:** Department of Genetics and Genome Biology, College of Life Sciences, University of Leicester, Leicester, United Kingdom

**Keywords:** bacteriophages, *Salmonella*, host range analysis, positive selection analysis, antibiotic resistance, phylogenetic analysis

## Abstract

A common cause of human food poisoning is through ingestion of pork products contaminated with *Salmonella* spp. Worryingly multi-drug resistant (MDR) *Salmonella* strains have been isolated from pigs, which motivates the need for alternative antimicrobials. In this study isolation and characterization of 21 lytic *Salmonella* phages is described. All 21 phages, labeled as SPFM phages were shown to efficiently infect MDR *Salmonella* strains isolated from United Kingdom pigs and phages SPFM1, SPFM3, SPFM10, SPFM14, SPFM15, SPFM17, and SPFM19 could lyse 100% of strains tested. The phage genome sizes range from 233 to 242 Kb, which qualifies them as jumbo phages. All SPFM phage genomes are approximately 95% similar to each other by average nucleotide identity, they encode between 258–307 coding sequences and share 188 core genes. Phylogenetic analysis shows these phages are most similar to phages of the genus *Seoulvirus* and to further characterize phages within the genus, genes under positive selection were identified. Several of the genes under evolutionary selection pressure were predicted to encode for proteins that interact with bacteria. We describe the phenotypic and genetic characterization of this novel *Salmonella* phage set. As the phages efficiently kill MDR *Salmonella* strains, they may offer a promising alternative to antibiotics.

## Introduction

Non-typhoidal *Salmonella* spp. is a leading cause of human food poisoning worldwide and responsible for 93 million infections annually (Torgerson et al., 2015). An estimated 11.7% of these infections are caused by the consumption of contaminated pork products (Pires et al., 2011). The most prevalent *Salmonella* serotypes associated with United Kingdom pigs and hence human infections are *S*. Typhimurium, *S*. 4:5:12:i:-, *S*. 4:5:12, and *S*. Derby, *S*. Bovismorbificans (Animal Health, and Veterinary Laboratories Agency, and Animal, and Plant Health Agency, 2014; Powell et al., 2016). Serotypes *S*. 4:5:12:i:- and *S*. 4:5:12 are antigenically and genetically similar to *S*. Typhimurium (Torgerson et al., 2015). Treating infections in pigs is becoming increasingly difficultand the number of infections caused by multi-drug resistant (MDR) *Salmonella* strains is growing (EFSA, 2017). It is clear that alternatives to antibiotics are urgently required to control *Salmonella* infection in pigs. Bacteriophages (phages) are viruses that target and kill bacteria and are one such alternative (Borie et al., 2014). Phages have a long history of being used as antimicrobials and there is an increasing interest in developing their use in animal husbandry due to their specificity, efficient bacterial lysis and their capability to self-replicate (Loc-Carrillo and Abedon, 2011; Nobrega et al., 2015). Phages that obligatory follow the lytic cycle, so lyse their target bacteria and are not capable of transduction, are considered to be optimal for therapeutic use (Pirnay et al., 2015).

Whole genome sequencing has made it easier to identify lytic phages, based on absence of known lysogeny modules and to characterize diversity within phages. From the completed *Salmonella* tailed phage genomes available on Genbank there is a huge variation in genome sizes from 53 to 250 Kb (Zhang et al., 2015). These *Salmonella* phages have been isolated from different environmental sources across the world, such as from sewage and faeces from chickens and pigs (Hooton et al., 2011; Moreno et al., 2013; Wongsuntornpoj et al., 2014; Bao et al., 2015; Hong et al., 2016). The majority of phages have genome sizes less than 200 Kb and to date only two *Salmonella* jumbo phages (with genomes larger than 200 Kb) have been described: SPN3US (Lee et al., 2011) and SEGD1. Both jumbo phages are related, and share common features with the well-described *Pseudomonas aeruginosa* jumbo phage PhiKZ and thus are referred to as PhiKZ-like jumbo phages (Krylov et al., 2007). They share a complex capsid structure that package enzymes in the virion for injection into the host upon infection. They also have extra genes responsible for nucleotide metabolism and genome replication, and encode additional proteins for lysis of bacterial cell-wall peptidoglycan in comparison to smaller genome sized phages (Lavysh et al., 2016; Thomas et al., 2016). Due to the large genome sizes of jumbo phages, many proteins have not been functionally characterized as they do not have counterparts in other phage genomes (Yuan and Gao, 2017). However, progress is being made in assigning functions to uncharacterized proteins and in a recent study on phage SPN3US, 11 hypothetical proteins were assigned a function by generating amber SPN3US mutants (Thomas et al., 2016; Weintraub et al., 2018). Another important feature of PhiKZ-like jumbo phages is they have two multisubunit RNA polymerases (RNAP): the first being virion RNAP, which is responsible for transcription of early genes and the second is non-virion RNAP that is involved in the transcription of late genes (Yuan and Gao, 2017). It has been shown experimentally that the presence of two multisubunit RNAP enables phage transcription to be less dependent on the host bacterial transcriptional machinery (Yakunina et al., 2015). Thus the presence of extra lysis genes and jumbo phages being less dependent on bacterial transcriptional machinery potentially makes them ideal candidates for use in phage therapy (Yuan and Gao, 2017).

Several phages have been reported to significantly reduce *Salmonella* colonization and shedding in experimental pig studies (Gebru et al., 2010; Callaway et al., 2011; Hooton et al., 2011; Saez et al., 2011). However, the phages presented as being effective, have generally not been well characterised. For example, minimal data is presented on host ranges, on their ability to target representative isolates of circulating pig associated *Salmonella* serotypes. Furthermore, not all of these phages used have been sequenced and the data used to predict if the phages are lytic. All of this information is extremely helpful when it comes to developing and licensing a natural-phage product (Henein, 2013; Pirnay et al., 2015). In addition, genome data can inform on our fundamental understanding of the biology of isolated phages, helps to ensure phages don’t encode toxins and to identify other genes of interest. The genome data can be compared to other sequenced phages to link genotype to phenotype (Pope et al., 2015). Specific gene evolution in phage genomes can also be monitored, like all genomes, those encoded by phages are naturally susceptible to random, non-synonymous changes (Gregory et al., 2016). If such gene changes are selected for, this is referred to as “positive selection.” Genes under positive selection are determined by having a high ratio of non-synonymous to synonymous nucleotide substitutions (dN/dS) (Booker et al., 2017). This is where genetic modification has led to changes in the phenotype of protein-coding genes and genes under positive selection are identified by using programs, which can compare homologous sequences and such comparative genomics data is needed (Bloom, 2017). By knowing which genes are under positive selection in the natural environment is both interesting *per se*, and could inform us further down the line which phage genes may be under positive selection and thus could potentially impact phage infectivity in a therapeutic context (Vincent et al., 2017).

In this work, we present phenotypic host range data and genome data for a group of jumbo *Salmonella* phages that have clear efficacy on United Kingdom pig associated *Salmonella* strains. Twenty-one phages, named SPFM phages were isolated from pig farms, boar faeces and from a food processing plant; all of which were collected in the United Kingdom. The SPFM phages were characterised and their virulence activity was tested against a large panel of prevalent and relevant circulating MDR United Kingdom -pig *Salmonella* strains isolated from outbreaks in United Kingdom farms. The efficiency of plating and host range were determined for the full phage set. SPFM phages were sequenced, compared to all previously sequenced *Salmonella* phages in the NCBI database (until December 2017), and extensively analyzed. This included determining their positively selected genes. This is the first study to fully characterise and sequence a large collection of jumbo phages that are targeted against United Kingdom-pig-associated *Salmonella* strains.

## Materials and Methods

### Bacterial Strains and Growth Conditions

In total 68 *Salmonella* strains were used in this study. *Salmonella enterica* subsp. enterica serovar Typhimurium SL1344 (accession number FQ312003) was used as a reference strain. The remaining 67 *Salmonella enterica* subsp. enterica strains, were isolated by the Animal and Plant health Agency (APHA) in Weybridge, United Kingdom from pigs in the United Kingdom between 2012 and 2015. From these strains the serotypes were: 22 *S*. Typhimurium; 15 *S*. 4,12:i:-; 10 *S*. 4,5,12:i:-; 10 *S*. Bovismorbificans and 10 *S*. Derby. All strains were resistant to at least one of the following antibiotics: Nalidixic acid, Tetracycline, Neomycin, Ampicillin, Furazolidone, Ceftazidime, Sulfamethoxazole Trimethoprim, Chloramphenicol, Amikacin, Amoxicillin/clavulanic acid, Gentamicin, Streptomycin, Compound Sulphonamide, Cefotaxime, Apramycin, and Ciprofloxacin.

All *Salmonella* strains were stored in 50% glycerol broth (Abtek Biologicals Ltd., United Kingdom) at -80°C. Strains were routinely grown on Xylose Lysine Deoxycholate (XLD) agar (Oxoid, United Kingdom) for 18 h at 37°C before being sub-cultured in NZCYM broth (Melford Biolaboratories Ltd., United Kingdom) for 18 h at 37°C at 100 rpm.

### Phage Isolation, Purification and Propagation

**Figure 1:**
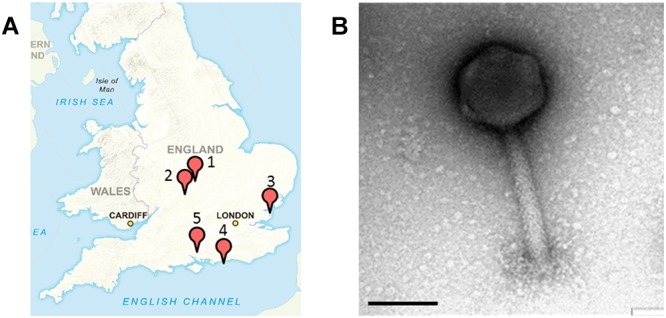
Sample sites in the United Kingdom where *Salmonella* SPFM phages were isolated and their phenotypic structures. **(A)** Multiple samples were sourced in the United Kingdom for phage isolation. (1) A nature reserve with wild boars in Hampshire (SPFM1 – SPFM3), (2) a food processing plant in Essex (SPFM4 – SPFM11), (3) pig farm in Warwick, Warwickshire (SPFM12 – SPFM15), (4) pig farm in Hinckley, Leicestershire (SPFM16 – SPFM20), and (5) a nature reserve with pigs in West Sussex (SPFM21 – SPFM22). United Kingdom map sourced with permission from Crown Copyright and Database Right (2017). Ordnance Survey (Digimap Licence). **(B)** All 21 isolated phages were identified by TEM as being myoviruses and a representative micrograph is presented. The black bar represents 100 nm.

All samples were collected from five geographical locations in the United Kingdom from July to December 2015. In total 15 samples were collected from a nature wild boar reserve in Hampshire; 10 samples from a food processing plant in Essex; 15 samples from a finishing pig farm in Warwickshire; 18 samples from pig farms in Leicestershire with piglets and finishing pigs; and seven samples from a nature reserve with wild pigs in West Sussex (Figure 1A).

All samples were processed using the same enrichment procedure, 1 ml or 1 g of sample was mixed with 9 ml of NZCYM broth and 100 μl of an exponential growing *Salmonella* culture was added. To maximise phage isolation the same sample was aliquoted and enriched with 12 different MDR *Salmonella* host strains individually. Enrichments were incubated at 37°C for 12 h with shaking (at 100 rpm), after which samples were centrifuged at 4 000 × *g* for 15 min at room temperature. The supernatant was filtered through 0.22 μm pore size syringe filters (Millipore, United Kingdom) and the filtered samples were stored at 4°C until further use. Filtrates were screened for phage by the small drop plaque assay method (Mazzocco et al., 2009). Briefly, 10 μl of enriched sample was spotted on a Luria-Bertani (LB) 1% (w/v) agar plate (Thermo Fisher Scientific, United Kingdom) with NZCYM 0.5% (w/v) agar as the top layer, which was mixed with 100 μl of exponentially growing *Salmonella* culture. Plates were incubated at 37°C for 18 h and examined for phage lysis either by presence of clearing or phage plaques.

For phage purification individual plaques was picked with 1 μl loops, mixed with 500 μl of SM buffer with gelatin (100 mM NaCl, 8 mM MgS0_4_.7H_2_O, 50 mM Tris–Hcl and 0.01% (w/v) gelatin) and centrifuged at 21,000 × *g* for 10 min. The resultant supernatant was used for the next round of phage purification by the double agar overlay plaque assay (Kropinski et al., 2009) and the process was repeated seven times to produce clonal phage stocks.

Increased volumes of purified phage lysates were made by mixing exponential growing liquid cultures of SL1344 (10^7^ CFU/ml) of *Salmonella* infected with 10^7^ PFU/ml phages in NZCYM broth at 37°C with shaking (100 rpm) for 6 h. Phage cultures were centrifuged at 4,200 × *g* for 15 min, the supernatant was filtered with 0.22 μm pore size filters and phage lysates were stored at 4°C. To determine phage titre, phage lysate was serially diluted 10-fold and the small drop plaque assay method (Mazzocco et al., 2009) was used on LB 1% agar plates. Final phage titres were expressed as PFU/ml.

### Transmission Electron Microscopy

Phage lysates were concentrated before Transmission electron microscopy (TEM) analysis by centrifugation at 21,000 × *g* for 1 h, the pellet was resuspended with 0.1 M ammonium acetate (Thermo Fisher Scientific, United Kingdom), centrifuged at 21,000 × *g* for 1 h and resuspended with a 0.1 M ammonium acetate solution. The highly concentrated phages (10^11^ PFU/ml) were negatively stained with 1% uranyl acetate (w/v) for 10 s and applied to 3 mm carbon coated copper grids (Agar Scientific Ltd., United Kingdom). Phages were examined with an EOL 1220 (JEOL UK Ltd., United Kingdom) ran at 80 kV and images were acquired by SIS Megaview III camera with analysis software (Olympus Soft Imaging Solutions, Germany) (Ackermann, 2009). TEM analysis was conducted by Dr. Ali Ali and Natalie Allcock, Core Biotechnology Services, University of Leicester, United Kingdom.

### Phage Host Range Analysis and Efficiency of Plating

The host range of individual phages was determined by the small drop plaque assay method (Mazzocco et al., 2009) on different *Salmonella enterica* subsp. enterica serotypes and incubated for 18 h at 37°C. Plates were examined for either bacterial lysis via clearing or plaques or for no infection and average observations were noted from three biological replicates, each with three technical repeats.

Efficiency of plating (EOP) was conducted on two representative MDR strains from five *Salmonella* serotypes: *S*. Typhimurium; *S*. 4,12:i:-; *S*. 4,5,12:i:-; *S*. Bovismorbificans and *S*. Derby. EOP was also established on the phages propagation host S1344. For EOP the small drop plaque assay method was used and phage lysates were 10-fold serially diluted and spotted onto bacterial lawns (Kutter, 2009). Average PFU/ml EOP values were calculated from three biological replicates each with three technical repeats. Principle component analysis was done for the EOP values of all phages, on all strains screened using the prcomp function in the R base package (R Core Team, 2017) and plotted as a biplot with the autoplot function from the ggplot2 package (Wickham, 2009).

### Phage DNA Extraction, Sequencing and Annotation

High titre phage lysate (10^10^ PFU/ml) was used to extract DNA using a revised phenol-chloroform-isoamyl method as previously described (Nale et al., 2016). Following the extraction the final DNA pellet was dissolved in 5 mM Tris–HCl, quantified using the Qubit fluorometer with the Qubit double-stranded HS kit (Thermo Fisher Scientific, United Kingdom) and sequenced by the Illumina MiSeq platform. NexteraXT libraries were prepared according to the manufacturer’s instructions, using 1 ng of input DNA. Reads were trimmed with Sickle v1.33 (Joshi and Fass, 2011) prior to assembly with SPAdes v v3.9.1 (Anton et al., 2012; Bankevich et al., 2012). Annotation was carried out with Prokka v1.11 (Seemann, 2014) using a custom database constructed from all phage proteins (December, 2017) and hmmscan to identify pVOGs (Grazziotin et al., 2017). The accession numbers assigned to the phage genomes are listed in Table 1. There was an error in uploading the phage genomes to European Nucleotide Archive (ENA) and the phage names are incorrect. Even so, the accession numbers listed in Table 1 are correct and allows to distinguish between the phages. ENA are currently resolving the issue and the correct files with the right names can be found via this link http://s3.climb.ac.uk/Sinfo/SPFM_genome.tar.gz.

**Table 1:** Summary of isolated phages with their respective isolation host, source in the United Kingdom and genome information.

Phage	Isolation host serotype	Source^a^	Genome size (bp)	Coding sequences (CDS)	tRNA	GC content (%)	Accession number^b^
SPFM1	*S*. Typhimurium	1	242,624	307	1	48.78	LR535901
SPFM2	*S*. 4,12:i:-	1	240,111	260	1	48.62	LR535921
SPFM3	*S*. Typhimurium	1	240,198	257	1	48.62	LR535920
SPFM4	*S*. Typhimurium	2	240, 197	257	1	48.62	LR535902
SPFM5	*S*. Typhimurium	2	240,194	287	1	48.88	LR535903
SPFM6	*S*. Typhimurium	2	240,198	305	1	48.62	LR535905
SPFM7	*S*. Typhimurium	2	240,197	290	1	48.62	LR535904
SPFM8	*S*. 4,12:i:-	2	240,197	297	1	48.84	LR535906
SPFM9	*S*. Typhimurium	2	240,197	298	1	48.62	LR535907
SPFM10	*S*. Typhimurium	2	240,197	285	1	48.62	LR535908
SPFM11	*S*. Typhimurium	2	240,197	257	1	48.62	LR535909
SPFM12	*S*. Typhimurium	3	240,197	305	1	48.62	LR535911
SPFM13	*S*. 4,12:i: -	3	241, 405	261	1	48.57	LR535910
SPFM14	*S*. 4,12:i: -	3	240,197	289	1	48.61	LR535912
SPFM15	*S*. Typhimurium	3	239,951	289	1	48.63	LR535913
SPFM16	*S*. 4,12:i: -	4	233,195	249	1	48.63	LR535915
SPFM17	*S*. 4,12:i: -	4	239,842	258	1	48.64	LR535914
SPFM19	*S*. 4,12:i: -	4	240,197	258	1	48.62	LR535916
SPFM20	*S*. Typhimurium	4	236,956	296	1	48.62	LR535917
SPFM21	*S*. Typhimurium	5	240,196	259	1	48.62	LR535919
SPFM22	*S*. Typhimurium	5	240,196	259	1	48.62	LR535918

^a^*Samples source numbers relate to Figure 1: (1) A nature reserve with wild boars in Hampshire, (2) a food processing plant in Essex, (3) pig farm in Warwick, Warwickshire, (4) pig farm in Hinckley, Leicestershire, and (5) a nature reserve with pigs in West Sussex. ^*b*^All genome sequences can be found via this link: http://s3.climb.ac.uk/Sinfo/SPFM_genome.tar.gz*.

### Calculating ANI Between *Salmonella* Phage Genomes

In total 158 genomes from *Salmonella*-infecting phages were collected from both phages newly sequenced in this study and phages sequenced from previous studies. The average nucleotide identity, as defined by Goris et al. (2007), was measured between all pairwise combinations of phage genomes using the BLASTN alignment option in the pyani package (Pritchard et al., 2016) and plotted in an interactive heatmap using heatmaply (Galili et al., 2017).

### Protein Ortholog Clustering

Phage protein sequences annotated by Prokka were clustered into orthologous groups using the COG triangles algorithm (Kristensen et al., 2010) implemented in the get_homologs.pl script (Vinuesa and Contreras-Moreira, 2015) using a BLASTP *e*-value threshold of 1e-5. The Jaccard distance between each phage genome was based on gene presence/absence as determined from COG clustering of phage protein sequences. The Jaccard distance was calculated using the base R dist function, a dendrogram was constructed from the distances using the base R hclust function, and the dendrogram was plotted using the ggdendro package in R (Vries and Ripley, 2016). Ortholog clustering data is shown in Supplementary Table 4.

### Postulating a Phylogenomic Tree Based on Core Genes

COGs that contained protein sequences from the *Seoulvirus* genus phage genomes were used to construct codon-aware alignments of the corresponding nucleotide sequences with MUSCLE v3.8.31 [Edgar, NAR 32(5)] and the pal2nal.pl script (Suyama et al., 2006). SNPs from the codon-aware nucleotide alignments were extracted with snp-sites (Page et al., 2016) and used to construct a phylogenomic tree using FastTree v.2.1.10 SSE3 (Price et al., 2010) with the generalized time-reversible model of nucleotide evolution.

### Measuring Positive Selection

A test for positive selection was done at each codon of each ortholog cluster using the HyPhy package (Pond et al., 2005). In particular, a protein alignment of each ortholog cluster from MUSCLE v3.8.31 was used to guide construction of a codon-aware alignment of the nucleotide sequences from each cluster. Recombination breakpoints in each codon-aware alignment were detected using the GARD algorithm (Pond et al., 2006) implemented in the HyPhy package and partitioned alignments and trees were constructed on either side of each breakpoint. These partitions were tested for positive selection at each site with the Bayesian FUBAR algorithm (Murrell et al., 2013) implemented in the HyPhy package, which returns the posterior probability that each site is under positive selection (that is, the dN/dS is greater than one). As the sample size is too small to allow accurate estimation of dN/dS at individual codons, the posterior probability values are given alongside the dN/dS values. The hypothesis test is sensitive enough to detect positive selection and the PSRF/N effective ratio was maintained below 0.006, so that the posterior probability of positive selection was converging on a solution.

## Results

### Phage Isolation, Plaque Morphology and Propagation

Samples for phage isolation were collected between July and December 2015 from a food processing plant, from wild and domestic pigs and boars in the United Kingdom. The 65 samples were screened and 21 phages were isolated, which are named SPFM1 to SPFM22. Phage SPFM18 was excluded from analysis due to its incomplete genome sequence. 15 SPFM phages were isolated on five different *Salmonella enterica* serotype Typhimurium strains and the remaining seven on *Salmonella enterica* serotype 4,12:i:- strains. Three phages originated from wild boar faeces, eight from the food processing plant, and ten from finishing pig and piglet faeces obtained from farms in Warwickshire, Hinckley and West Sussex (Figure 1A and Table 1).

In terms of plaque morphology, phages SPFM9, SPFM10, and SPFM11 were isolated on *S.* Typhimurium strains and produced clear ∼1 mm in diameter plaques and the remaining 18 phages produced clear plaques of ∼0.5 mm in diameter. All phages were propagated on *S.* Typhimurium SL1344 to produce high titre [10^10^ plaque forming units (PFU)/ml] stocks. The main motivation for this is that the original isolation strains spontaneously release prophages, which complicates downstream characterization, whereas prophage release from SL1344 was never observed.

### Phage Morphology

Transmission electron microscopy analysis revealed the 21 phages have isometric heads and contractile tails and so were classed as members of the *Myoviridae* family within *Caudovirales* (Figure 1B). Phages SPFM1, SPFM2, SPFM6, SPFM7, SPFM10, SPFM12, SPFM14, SPFM15, SPFM16, SPFM17, SPFM20, and SPFM22 have tail lengths of 160 ± 20 nm and capsid diameters of 100 ± 15 nm. Phages SPFM3, SPFM4, SPFM5, SPFM8, SPFM9, SPFM11, SPFM19, and SPFM21 have tail lengths of 200 ± 20 nm and capsid diameters of 105 ± 15 nm. Phage SPFM13 had the longest tail at 140 ± 10 nm and a capsid diameter of 135 ± 7 nm.

### Host Range Analysis

**Figure 2:**
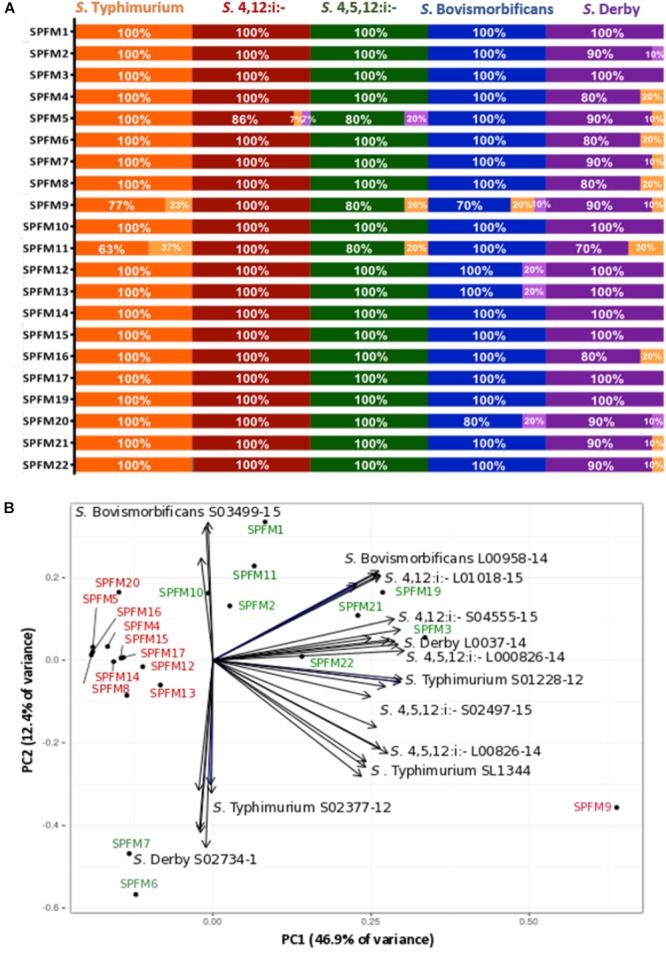
Host range analysis of 21 *Salmonella* SPFM phages and their efficiency of plating. **(A)** Host range analysis was based on the lysis profile of SPFM phages, against multi-antibiotic resistant strains: 22 *S*. Typhimurium; 15 *S*. 4,12:i:-; 10 *S*. 4,5,12:i:-; 10 *S*. Bovismorbificans and 10 *S*. Derby and complete lysis is presented by orange, red, green, blue, and purple bars, respectively. Turbid clearing is shown in light orange bars and no infection in light purple. **(B)** Efficiency of plating of SPFM phages on 11 *Salmonella* strains, including the phages propagation host SL1344 and data presented was analyzed by principle component analysis. The bioplot represents two principle components that contain the most variance (59.3% variance in total) for EOP of SPFM phages (labeled black circles) on two representative strains from five pig-associated *Salmonella* serotypes and EOP on the phage’s propagation host *S*. Typhimurium SL1344 (11 strains in total). Phages are colored depending on their EOP, where phages in red are those that show the same EOP on all strains and in green are phages that have higher EOP on specific strains and are positioned closer to individual strains. For both host range analysis **(A)** and EOP analysis **(B)** three biological replicates were conducted and data presented is the average of all three.

The efficacy of SPFM phages was tested on 67 MDR *Salmonella enterica* subsp. enterica strains isolated from pigs. All 67 strains are representatives of the top five United Kingdom pig associated *Salmonella* serotypes, namely *S*. Typhimurium, *S*.4,12:i:-, *S*.4,5,12:i:-, *S*. Bovismorbificans, and *S*. Derby. All phages have a wide lytic spectrum of activity, and each lyses over 80% of strains (Figure 2A). Phages SPFM1, SPFM3, SPFM10, SPFM14, SPFM15, SPFM17, and SPFM19 could lyse 100% of *Salmonella* strains screened. Phages SPFM2, SPFM7, SPFM21, and SPFM22 lysed 67/68 of strains (99%). SPFM9 and SPFM11 in contrast only lyse 84 and 81% of strains, respectively, and even where they lyse bacteria, lysis is turbid on at least one strain.

### Efficiency of Plating (EOP)

In order to determine how efficient phage infection is on clinically relevant strains, and thus decide which phages should ultimately make up a phage cocktail, the EOP for the SPFM phages was carried out on two representative MDR pig strains from *Salmonella* serotypes *S*. Typhimurium, *S*.4,12:i:-, *S*.4,5,12:i:-, *S*. Bovismorbificans, and *S*. Derby. The EOP of phages on their propagation host, *S*. Typhimurium SL1344 was included as a control. To identify patterns within the EOP data set a principle component analysis (PCA) was used, which revealed that the phages cluster into two distinctive groups (Figure 2B); (i) phages that have similar EOP on all strains and (ii) phages that have higher EOP on specific *Salmonella* strains and serotypes. Phages SPFM4, SPFM5, SPFM8, SPFM9, SPFM12, SPFM13, SPFM14, SPFM15, SPFM16, SPFM17, and SPFM20 formed group (i) and their EOP were not significantly different among all five serotypes, confirmed by *T*-tests. The remaining 10 phages formed group (ii). To expand on group (ii) phage SPFM10 had a higher EOP on *S*. Bovismorbificans isolate A; phages SPFM1, SPFM11, and SPFM12 on all *S*. Bovismorbificans strains. Phages SPFM19 and SPFM21 had higher EOP on both *S*.4,12:i:- strains; SPFM3 on *S*. Derby isolate B, SPFM22 on *S*. 4,5,12:i:- isolate A and both phages SPFM6 and SPFM7 on *S*. Derby isolate A.

### Genome Characterization of SPFM Phages

The SPFM phage set was sequenced using the Illumina MiSeq platform. All 21 phages have linear, circularly permuted dsDNA genomes ranging from 233 to 242 Kb and encode between 258 and 307 coding sequences (CDS) (Table 1). As all genomes are larger than 200 Kb they are classified as jumbo phages and all are predicted to be lytic based on the absence of known lysogeny associated genes.

**Figure 3:**
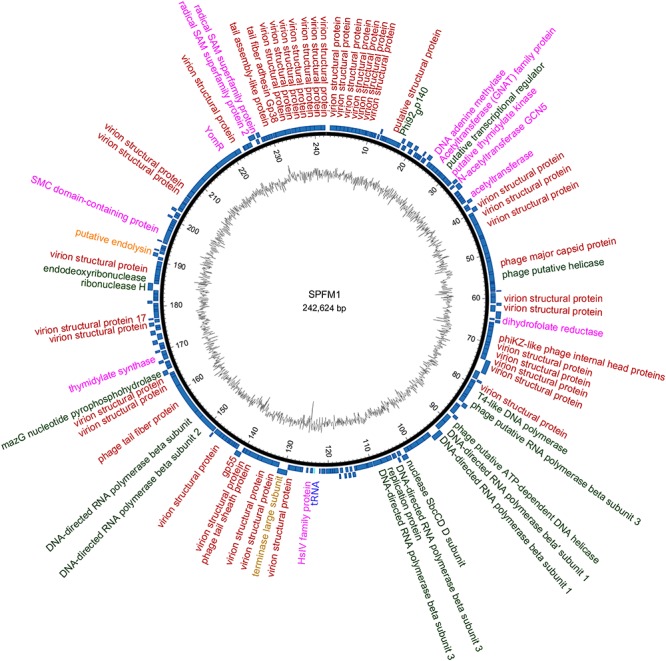
An example representation of SPFM phages and presented is the genome of phage SPFM1. The inner black circle shows the GC skew and the scale units are base pairs. The inner blue circle represents the predicted open reading frames in forward strand and the predicted open reading frames in the reverse strand are shown in the outer circle. Functional genes belonging to different categories are colored accordingly: in red are structural proteins, in green are genes involved in DNA replication and transcription, in yellow are packaging genes, orange are lysis genes, highlighted in purple are additional genes and the tRNA is labeled in blue. Unlabeled ORFs represent hypothetical proteins. The genome was drawn using the software Circos.

Despite the variation in SPFM genome size, they all encode one tRNA, have average GC contents of 48.5% (Table 1), an average gene length of 0.860 ± 0.010 Kb, gene densities of 1.074/kb and gene coding regions constitutes 93% of their genomes. As the phages are genetically similar in architecture as well as content, a representative genome map of SPFM1 is shown in Figure 3. The majority of the predicted genes encode proteins with no known function and putative roles could only be assigned to ∼30% of genes. Genes recognizable by homology to other phages include those that encode for structural proteins, such as the major capsid proteins, a tail fiber protein and a tail sheath protein. The gene encoded for the packaging protein terminase was also identified, as was the phage endolysin. Several genes encoding products involved with DNA replication and transcription could be identified such as endodeoxyribonuclease, helicase, putative nuclease SbcCD D subunit, putative ribonuclease H and six RNAP beta (ββ/β’) multisubunits (Supplementary Table 1). The genes encoding RNAP multi-subunits vary in length from 0.240 to 4.206 Kb and all six subunits had 99, 80, and 55% average nucleotide identity (ANI) to *Salmonella* SPN3US (accession number: JN641803.1), *Erwinia* phage vB (accession number: KX397364.1) and *Cronobacter* phage CR5 (accession number: JX094500) (Lee et al., 2016), respectively.

Other genes were identified in all SPFM phage genomes, which could potentially alleviate their dependency on their bacterial host during infection. Genes of particular interest include dihydrofolate reductase, thymidylate synthase and thymidylate kinase, all of which are predicated to be used for folate synthesis and radical *S*-adenosylmethionine (SAM) genes involved in enhancing host metabolism during phage infection (Lee et al., 2011). In addition, DNA adenine methylase gene was identified in SPFM phages that could provide defence against the hosts’ restriction modification systems.

### Hierarchical Cluster Analysis of Isolated SPFM Phages

**Figure 4:**
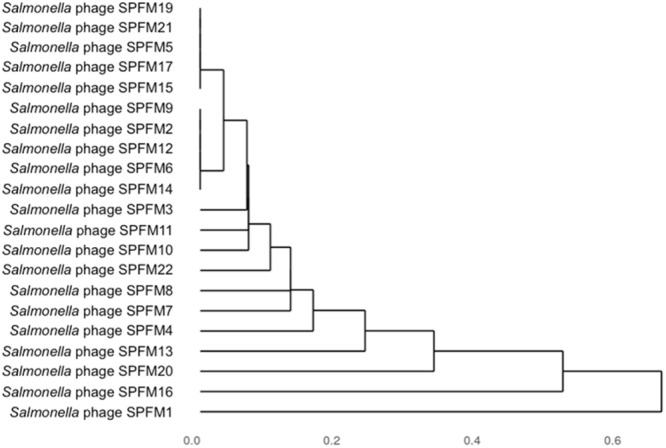
Dendrogram of 21 *Salmonella* SPFM phages generated from hierarchical cluster analysis based on accessory genes presence and absence. Jaccard distances, a measure of dissimilarity between the phages were calculated based on presence and absence data for 46 accessory genes and hierarchically clustered. The unit 0 shows the presence of identical genes and 1 showing the most variation.

Genomes were compared using pairwise local alignment with nucleotide BLAST (NCBI, 1988) and it was observed all SPFM phages were genetically very similar to each other with ∼95% ANI. To gain an insight into variation within the genomes a dendrogram was built based on the presence or absence of 46 shared accessory genes (Figure 4). The hierarchical cluster analysis identified phages SPFM5, SPFM15, SPFM17, SPFM19, SPFM21 were most similar to each other. Phages SPFM2, SPFM6, SPFM9, SPFM12, and SPFM14 formed a second group and shared the same accessory genes and core genes. The remaining eleven phages demonstrated variation between the accessory genes and the most differences were between phages SPFM1, SPFM13, SPFM16 and SPFM20. All of which also had differences in their genome sizes in comparison to the other SPFM phages (Table 1).

### Comparison of SPFM Phages to Previously Sequenced *Salmonella* Phages

**Figure 5:**
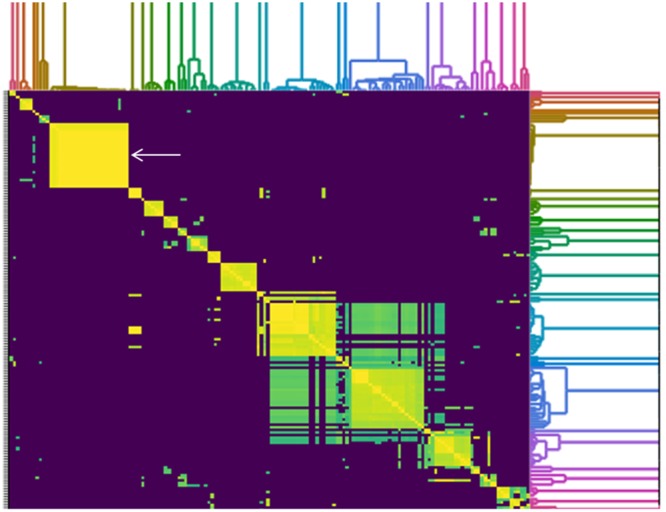
Heatmap of pairwise average nucleotide identity (ANI) values for 158 whole genome sequenced *Salmonella* phages in the NCBI database (until December 2017), including 21 SPFM phages from this study. Values range from 0 (0%) ANI to 1 (100% ANI): purple represents 0% ANI, clusters of highly similar phages are highlighted in yellow and green and the colored branches represent different clusters. The SPFM phages all cluster together on the heatmap and the cluster is positioned on the upper right (solid yellow box, indicated by the white arrow). All phages used to construct heatmap are listed in Supplementary Table 2.

To determine how similar SPFM phages are to previously sequenced *Salmonella* phages deposited in Genbank (until December 2017), an all-versus-all comparison analysis against 158 fully sequenced *Salmonella* phage genomes was conducted (Figure 5). The genome sizes of all *Salmonella* phages used for the analysis ranged from ∼33 to 240 Kb and all the phages used in the analysis are listed in Supplementary Table 2. A cluster analysis identified 21 distinct groups, where a cluster is defined as phages sharing >50% of their ANI with other members of the cluster. All 21 SPFM phages group together in one cluster with phages SPN3US (accession number: JN641803.1) (Lee et al., 2011) and SEGD1 (accession number: KU726251.1) isolated in different studies. Phages SPN3US and SEGD1 also have genomes of ∼240 Kb and have ∼95–97% ANI with all SPFM phages. The phages SPN3US and SEGD1 are part of the *SPN3USvirus* genus (Adriaenssens et al., 2017), which has now been updated to *Seoulvirus* genus. This is based on the current standards of ANI above 95% and the SPFM phages also fall into this genus. The phages within the *Seoulvirus* genus cluster are classed as phiKZ-like phages.

### *Seoulvirus* Genus Phage Cluster

To determine how closely related the SPFM phages are to the *Salmonella* phages SPN3US and SEGD1, a phylogenetic analysis was constructed (Supplementary Figure 1) based on single nucleotide polymorphisms (SNPs) within the shared 188 core genes (Supplementary Table 3). This revealed that phages SPFM5, SPFM9, SPFM10, and SPFM11 share the same SNPs. This clustering of phages according to SNPs in the core genes differs from the hierarchical cluster analysis based on the presence or absence of accessory genes. The other phages (apart from SPFM1), grouped together and had little or no variation in their core genes. SNPs of SPFM1 core genes caused this phage to group as a separate clade from the other 20 SPFM phages. Phages SPN3US and SEGD1 formed a tighter subclade distinct from the SPFM phages (Figure 4).

### Phage Genes Under Positive Selection

To determine which genes are under evolutionary selection pressure, genes under positive selection were identified within the genus *Seoulvirus* cluster. This was carried out by determining the ratio of non-synonymous over synonymous substitution rates (dN/dS) of pairwise comparison of core orthologs of the 21 SPFM phages, SPN3US and SEGD1 (Supplementary Table 5). The data presented in Table 2 illustrates the posterior probability values above >0.900 for the predicted genes under positive selection and their ortholog cluster number. The analysis predicted that 33 genes are under positive selection. 22 of these genes are putative virion structural proteins, one is a putative endodeoxyribonuclease RusA, two encode thymidylate synthase and eight are hypothetical proteins. For the putative virion structural proteins under positive selection, HHpred (Zimmermann et al., 2017) was used to determine if the structural proteins are involved in phage tail or capsid assembly. The program predicted with over 75% probability that they were putative baseplate wedge proteins and thus likely to be involved in phage tail assembly. In addition, the ortholog cluster number 201 (relates to the ortholog cluster number in Supplementary Table 5) had 98.26% probability hits to C-terminal pectate lyase domain, which is also part of phage tail fiber formation. Not all genes under positive selection could be assigned a function but it can be predicted the following hypothetical proteins with ortholog cluster numbers 106, 108, 129, 130 and 211 could potentially be putative virion structural proteins due to their localization close to other putative structural proteins on the genome. Similarly, the hypothetical protein with ortholog cluster number 92 is positioned close to where the DNA replication and transcription genes are clustered and so likely involved in this function.

**Table 2:** Genes under positive selection within the *Seoulvirus* phage cluster, which includes phages SPN3US, SEGD1, and 21 SPFM phages.

Ortholog cluster number^a^	Cluster predicted protein	Reannotation of predicted protein^b^	Codon number	Posterior probability (dN/dS)^c^	dN/dS omega value^c^
92	Hypothetical protein		627	0.9098	0.1179
100	Putative virion structural protein		553	0.9223	0.0648
100	Putative virion structural protein	Putative baseplate wedge protein	762	0.9192	0.0648
106	Hypothetical protein		253	0.9332	0.1172
106	Hypothetical protein		246	0.9019	0.3001
108	Hypothetical protein		55	0.9404	0.2553
108	Hypothetical protein		35	0.9402	0.2553
109	Putative virion structural protein		110	0.9462	0.284
129	Hypothetical protein		200	0.9479	0.0417
130	Hypothetical protein		62	0.9697	0.212
139	Putative endodeoxyribonuclease RusA		209	0.9397	0.2496
190	Putative virion structural protein	Putative DNA binding protein	226	0.9545	0.1129
192	Putative virion structural protein		39	0.9463	0.4287
192	Putative virion structural protein		41	0.9449	0.4545
192	Putative virion structural protein		73	0.9265	0.4287
194	Putative virion structural protein		207	0.9315	0.1397
196	Putative virion structural protein		337	0.9448	0.4848
196	Putative virion structural protein		421	0.9333	0.2123
196	Putative virion structural protein		124	0.9033	0.237
199	Putative virion structural protein		429	0.9469	0.0105
201	Putative virion structural protein	Putative pectate lyase domain	271	0.9783	0.3591
204	Putative virion structural protein		210	0.9143	0.6042
205	Putative virion structural protein		292	0.9244	0.2598
211	Hypothetical protein		27	0.9413	0.1693
219	Putative virion structural protein		241	0.9167	0.1225
222	Putative virion structural protein	Putative baseplate wedge protein	981	0.9272	0.1589
222	Putative virion structural protein	Putative baseplate wedge protein	314	0.9190	0.1806
223	Putative virion structural protein	Putative short tail fiber	207	0.9747	0.2195
234	Putative virion structural protein		38	0.9253	0.001
281	Thymidylate synthase		89	0.9236	0.1395
281	Thymidylate synthase		92	0.9019	0.0625
289	Putative virion structural protein		118	0.9018	0.0805
302	Putative virion structural protein		72	0.9652	0.9065

^a^*Ortholog cluster numbers relate to Supplementary Tables 4, 5. Raw dN/dS data is presented in Supplementary Table 5. ^*b*^The tool HHPred (Zimmermann et al., 2017) was used to reannotate protein sequences and presented are annotations with over 75% probability. ^*c*^The sample size is too small to allow accurate estimation of dN/dS at individual codons, but the hypothesis test (the posterior probability) is sensitive enough to detect positive selection*.

## Discussion

Several studies have shown that phages can significantly reduce the amount of *Salmonella* spp. bacteria from food settings and in pigs, which suggests that phages are a viable tool to improve food safety (Zhang et al., 2015). However, to date most published studies do not characterize their phages, based on their efficacy to lyse target strains or by sequencing. In this study we aim to address this and we have fully characterized 21 new *Salmonella* phages. All phages could infect representative MDR strains isolated from pigs and to our knowledge this is the largest host range analysis conducted on phages that target pig associated *Salmonella* strains. Seven virulent phages were identified that could infect 100% of *Salmonella* isolates tested. A possible explanation of the high infectivity of these phages could be that as the phages infect *S*. Typhimurium, they can also infect its monophasic variants, which are genetically closely related (Moreno et al., 2013). It is likely the phages are using the same bacterial receptor to attach to strains from these serotypes. These seven phages appear to be ideal for therapeutic use based on their host range and efficiency of plating. Broad-host range *Salmonella* phages have been reported in the literature (O’Flynn et al., 2006; Sillankorva et al., 2010; Santos et al., 2011; Pereira et al., 2016). However, no studies have presented data on phage(s) being able to lyse all *Salmonella* strains associated with pigs.

Two phages SPFM9 and SPFM11 could only infect ∼80% of the strains screened. Additionally, both phages produced turbid clearing on a fifth of the strains, which could indicate potential lysogeny as turbid clearing is often a characteristic of temperate phages (Gallet et al., 2011). Although, sequence analysis confirmed both phages had no known lysogeny modules and are likely to be lytic phages but as only ∼30% of genes have been assigned with a known function, unknown lysogeny modules could exist. A possible explanation to why turbid clearing was observed could be that the bacterial strains screened may have been partly resistant to the phage so only a sub population of cells were infected, which resulted in turbid clearing (Bull et al., 2014). An alternative explanation could be the phages are inducing a prophage within these strains, which could have produced the turbid clearing (Campoy et al., 2006). Although turbid clearing was observed, SPFM9 and SPFM11 were able to infect and replicate on the 11 strains screened for EOP analysis, which indicates lytic infection. Due to potentially incomplete lysis and issue of resistance or induction of prophages, phages SPFM9 and SPFM11 would not be good candidates for therapy (Chan et al., 2013; Abedon et al., 2017).

To further characterize SPFM phages and to narrow down which have ideal traits for use therapeutically, EOP analysis was conducted (Mirzaei and Nilsson, 2015). The data revealed a group of phages that had no differences in EOP across all representative strains from five different serotypes and as there was no difference in infectivity between the strains, these phages would appear to be ideal candidates for therapeutic application. In particular phages SPFM14, SPFM15, and SPFM17 are good candidates, as they could infect all representative strains from the dominant United Kingdom *Salmonella* serotypes and had high EOP’s on the strains screened.

All isolated SPFM phages had genomes greater than 233 Kb and will significantly add in numbers and diversity to the ∼170 jumbo phage genomes available on NCBI (Yuan and Gao, 2017). The genomes of the SPFM phages were also bigger in size in comparison to other *Salmonella* phages, such as the myovirus vB_SalM_SJ_3 that has a genome size of 162,910 bp (Wall et al., 2010; Zhang et al., 2010, 2014; Saez et al., 2011) and the podovirus UAB_78 that has a genome size of 48,110 bp (Bardina et al., 2016). Jumbo phages are rare to isolate and are not frequently isolated by conventional methods, which can be biased toward smaller genome size phages (Serwer et al., 2009; Salmond and Fineran, 2015; Hillyard et al., 2016; Saad et al., 2018). However, it can argued, from the total phages described to date, approximately 2% are jumbo so it could be jumbo phages are truly rare as stated previously and are not actually underrepresented (Yuan and Gao, 2017). Especially as, in this study conventional methods were used for phage isolation (Twest and Kropinski, 2009; Yuan and Gao, 2017). It could be speculated that SPFM jumbo phages are well distributed, genetically stable and/or endemic in the United Kingdom, in different ecological locations in their natural settings (Angly et al., 2009). We plan to capitalize on these jumbo phages to develop a phage cocktail targeted against *Salmonella* serotypes associated with pigs.

All SPFM phages were genetically similar to each other, even though different *Salmonella* strains were used for enrichment and samples from a variety of environmental sources were collected (Jurczak-Kurek et al., 2016). The SPFM phages do differ in SNP’s, which could be present in host-interacting proteins. This could affect the attachment kinetics of the phage to the cell surface, leading to changes in host specificity (Switt et al., 2013). This potentially could explain why differences were observed in host range and EOP between the SPFM phages. A similar observation was described with 90% genetically identical *Pseudomonas* phages and likewise SNP’s lead to phenotypic differences between the phages (Ceyssens et al., 2011). It should be noted that phages SPFM5, SPFM9, SPFM10, and SPFM11 share the same SNPs, which could suggest they represent clones with SNP’s. The SNPs could have been induced by the propagation host, which was different from the host the phages were originally isolated on. To understand the significance of SNP’s in the core genes of SPFM phages, further analysis and mutation studies need to be conducted. Furthermore studies could include propagation of the phages in the same host for several generations to observe differences of genes under positive selection and SNPs and assess for a possible convergence.

SPFM phages were compared to all sequenced *Salmonella* phages, and they clustered with other known jumbo *Salmonella* phages SPN3US (Lee et al., 2011) and SEGD1, isolated in South China from chicken feces and in South Korea, respectively. It is very interesting that the SPFM phages where isolated in the United Kingdom but cluster and are genetically similar to phages isolated in a different continent. Similarly 87% genetically similar *Pseudomonas* phages were isolated from different countries in the United States and Europe (Ceyssens et al., 2011). Unfortunately, both phages SPN3US and SEGD1 have not been characterized in terms of host range and EOP, preventing direct phenotypic comparison with phages in this study. Furthermore the genome of SEGD1 was only deposited and no further characterisation of the phage has been conducted.

The large genome sizes of jumbo phages, allows for the carriage of numerous genes not present in smaller genome sized phages (Yuan and Gao, 2017), such as the six RNAP beta subunits that all SPFM phages have. The multiple RNAP subunits of phage SPN3US have been extensively studied in recent publications (Hillyard et al., 2016; Ali et al., 2017) and are very similar to RNAP beta subunits of phiKZ-like phages (Krylov et al., 2007; Lavysh et al., 2016; Yutin et al., 2018). A further three RNAP subunits were predicted recently in phage SPN3US by the construction of amber mutants of phage genes (Thomas et al., 2016; Ali et al., 2017). All three predicted subunits were also identified in SPFM phages: nvRNAP β^′^, vRNAP β present in the C terminus and vRNAP β^′^ present in the C terminus (Supplementary Table 1). Furthermore, the predicted three RNAP subunits are part of the core genes shared between the phages. Presence of multiple RNAP beta subunits is consistent with other sequenced jumbo phages, such as the seven RNAP beta subunits that have been identified bioinformatically in *V. coralliilyticus* phage BONAISHI (Jacquemot et al., 2018); *Ralstonia solanacearum* phages RP12 and Φ8RP31 (Matsui et al., 2017). Overall the presence of extra genes in jumbo phages could reduce their dependence on their bacterial host for essential proteins associated with the phage lifecycle and consequently could help broaden the phage host range (Yuan and Gao, 2017). This could explain why all SPFM phages can infect multiple clinically relevant *Salmonella* isolates, which could make SPFM phages ideal candidates for phage therapy.

Within the *Seoulvirus* genus, genes under positive selection were identified from the core genes shared by the phages. Genes under positive selection included host-interacting proteins, such as two putative virion structural proteins predicted to be baseplate wedge proteins and involved in the formation of tail fibers, both of which are involved in binding of phages to bacterial cells. These host-interacting proteins have to adapt to different bacterial hosts, which could explain why they are under positive selection. These results could also give a rational explanation in the differences observed in host range and EOP between the SPFM phages. Other phage studies have also identified host-interacting proteins as being under positive selection, such as gene gp6 that encodes the baseplate protein and likely to be involved in host specificity (Vincent et al., 2017). Further genetic and mutation studies are needed to characterize phage genes under positive selection to understand their importance.

This study has described and characterized 21 genetically similar lytic jumbo phages that can lyse *Salmonella* strains commonly associated with United Kingdom pigs. Comprehensive host range analysis and EOP identified a number of phages that would be ideal candidates for phage therapy to improve food safety. Further work will focus on identifying the best phage cocktails that can maximally reduce *Salmonella* both *in vitro* and *in vivo* and determining the optimal delivery method of phages to pigs.

## Patents

The phages are part of a Leicester patent, pending. United Kingdom Patent Application 1815483.1.

## Data Availability

The datasets generated for this study can be found in ENA, LR535901.

## Author Contributions

AT and MC designed the experiments and drafted the manuscript. AT isolated the phages and conducted the host range analysis. AM sequenced all the phages. AT and NB analyzed and interpreted the data. NB, AM, and MC edited the manuscript. All authors agreed to be accountable for all aspects of the manuscript and approved the final version to be published.

## Conflict of Interest Statement

The authors declare that the research was conducted in the absence of any commercial or financial relationships that could be construed as a potential conflict of interest.
