# Supplementary material for: Genomic Characterization of Jumbo Salmonella Phages That Effectively Target United Kingdom Pig-Associated Salmonella Serotypes
Source: Front Microbiol. 2019 Jul 2;10:1491. doi: 10.3389/fmicb.2019.01491 (PMC6614189; doi:10.3389/fmicb.2019.01491)
Supplement: Supplementary file 1 [file Table_1.DOCX]

Supplementary Material

# Supplementary Figures and Tables

**Supplementary Figure 1.** Phylogenomic tree displays the relationship between 21 SPFM phages and phages SEGD1 and SPN3US within the genus *SPN3USvirus*. The tree was built from single nucleotide polymorphisms (SNPs) taken from codon alignments of 188 core genes shared by all the 23 sequenced phages with the generalized time-reversible model of nucleotide evolution. The tree is drawn to scale and the scale bar represents the relative genetic distances.

**Supplementary Table 1**. Viral (v) RNAP and non-viral (nv) RNAP subunits present in all SPFM phages when compared to annotated and predicted RNAP’s in phage SPN3US.

|  | Gene product in SPN3US genome^1^ | Protein ID in SPN3US genome^1^ | Annotation^2^ |
| --- | --- | --- | --- |
| nvRNAP subunits | 23 | [YP_009153317.1](https://www.ncbi.nlm.nih.gov/protein/849122557) | nvRNAP β’ |
|  | 30 | [YP_009153324.1](https://www.ncbi.nlm.nih.gov/protein/849122564) | Predicted nvRNAP |
|  | 34 | [YP_009153328.1](https://www.ncbi.nlm.nih.gov/protein/849122568) | nvRNAP β |
|  | 35 | [YP_009153329.1](https://www.ncbi.nlm.nih.gov/protein/849122569) | nvRNAP β’ |
|  | 77 | [YP_009153371.1](https://www.ncbi.nlm.nih.gov/protein/849122611) | Predicted nvRNAP β’ |
| vRNAP subunits | 42 | [YP_009153336.1](https://www.ncbi.nlm.nih.gov/protein/849122576) | vRNAP β’C |
|  | 218 | [YP_009153342.1](https://www.ncbi.nlm.nih.gov/protein/849122582) | Predicted vRNAP βC |
|  | 240 | [YP_009153534.1](https://www.ncbi.nlm.nih.gov/protein/849122774) | vRNAP β’N |
|  | 241 | [YP_009153535.1](https://www.ncbi.nlm.nih.gov/protein/849122775) | vRNAP βN |
|  | 244 | [YP_009153538.1](https://www.ncbi.nlm.nih.gov/protein/849122778) | Predicted vRNAP β’C |

^1^ Gene product, protein ID and annotations are from SPN3US genome with GenBank number JN641803.1 and publications (Ali *et al.*, 2017; Thomas *et al.*, 2016).

^2^ C, C terminus region and N, N Terminus region.

**Supplementary Table 2**. List of all *Salmonella* phage genomes used to construct the heatmap in Figure 5 and their order in the heatmap.

| Phage ID | Accession number |
| --- | --- |
| *Salmonella*_phage_Fels-1 | NC_010391 |
| *Salmonella*_phage_SEN4 | NC_029015 |
| *Salmonella*_phage_RE-2010 | NC_019488 |
| *Salmonella*_phage_ST64B | NC_004313 |
| *Salmonella*_phage_118970_sal3 | NC_031940 |
| *Salmonella*_phage_Fels-2 | NC_010463 |
| *Salmonella*_phage_SEN8 | KT630647.2 |
| *Salmonella*_phage_SEN1 | NC_029003 |
| *Salmonella*_virus_PsP3 | NC_005340 |
| *Salmonella*_phage_FSL_SP-004 | NC_021774 |
| *Salmonella*_phage_SSE121 | NC_027351 |
| *Salmonella*_phage_PVP-SE1 | NC_016071 |
| *Salmonella*_phage_38 | NC_029042 |
| *Salmonella*_phage_40 | KR296694 |
| *Salmonella*_phage_21 | NC_029050 |
| *Salmonella*_phage_vB_SenS_Sergei | KY742649 |
| *Salmonella*_phage_vB_SenS_Sasha | KX987158 |
| *Salmonella*_phage_9NA | NC_025443 |
| *Salmonella*_phage_vB_SalM_SJ2 | NC_023856 |
| *Salmonella*_phage_STML-13-1 | JX181828 |
| *Salmonella*_phage_ViI | FQ312032 |
| *Salmonella*_phage_Maynard | NC_022768 |
| *Salmonella*_phage_Marshall | NC_022772 |
| *Salmonella*_phage_SFP10 | NC_016073 |
| *Salmonella*_phage_GG32 | NC_031045 |
| *Salmonella*_phage_PhiSH19 | NC_019530 |
| *Salmonella*_phage_vB_SalM_PM10 | NC_031128 |
| *Salmonella*_phage_vB_SalM_SJ3 | NC_024122 |
| *Salmonella*_phage_Det7 | NC_027119 |
| *Salmonella*_phage_SKML-39 | NC_019910 |
| *Salmonella*_phage_FSL_SP-076 | NC_021782 |
| *Salmonella*_phage_FSL_SP-058 | NC_021772 |
| *Salmonella*_phage_FSL_SP-101 | KC139511 |
| *Salmonella*_phage_LSPA1 | NC_026017 |
| *Salmonella*_phage_Jersey | NC_021777 |
| *Salmonella*_phage_SETP13 | NC_022752 |
| *Salmonella*_phage_SETP7 | NC_022754 |
| *Salmonella*_phage_vB_SenS_AG11 | JX297445 |
| *Salmonella*_phage_BPS11Q3 | NC_031925 |
| *Salmonella*_phage_LPSE1 | KY379853 |
| *Salmonella*_phage_MA12 | NC_031021 |
| *Salmonella*_phage_L13 | NC_021317 |
| *Salmonella*_phage_wksl3 | JX202565 |
| *Salmonella*_phage_SS3e | NC_006940 |
| *Salmonella*_phage_SE2 | NC_016763 |
| *Salmonella*_phage_vB_SenS-Ent3 | NC_024204 |
| *Salmonella*_phage_Ent1 | HE775250 |
| *Salmonella*_phage_vB_SenS-Ent2 | NC_023608 |
| *Salmonella*_phage_SETP3 | NC_009232 |
| *Salmonella*_phage_f3SE | KU951147 |
| *Salmonella*_phage_f2SE | KU951146 |
| *Salmonella*_phage_f18SE | NC_028698 |
| *Salmonella*_phage_fSE4S | KT881477 |
| *Salmonella*_phage_fSE1C | KT962832 |
| *Salmonella*_phage_FSL_SP-031 | [NC_021775](https://www.genome.jp/dbget-bin/www_bget?refseq:NC_021775) |
| *Salmonella*_phage_64795_sal3 | [NC_031918](https://www.genome.jp/dbget-bin/www_bget?refseq:NC_031918) |
| *Salmonella*_phage_Vi_II-E1 | NC_010495 |
| *Salmonella*_phage_BP12B | [NC_031271](https://www.genome.jp/dbget-bin/www_bget?refseq:NC_031271) |
| *Salmonella*_virus_SP6 | NC_004831 |
| *Salmonella*_phage_SE1 | NC_011802 |
| *Salmonella*_phage_ST160 | NC_014900 |
| *Salmonella*_phage_ST64T | AY052766 |
| *Salmonella_*phage_g341c | NC_013059 |
| *Salmonella*_phage_vB_SemP_Emek | JQ806763 |
| *Salmonella*_phage_SPN9CC | NC_017985 |
| *Salmonella*_phage_vB_SosS_Oslo | NC_018279 |
| *Salmonella*_phage_SEN22 | NC_028696 |
| *Salmonella*_phage_34 | EU570103 |
| *Salmonella*_phage_25 | KR296687 |
| *Salmonella*_phage_P22-pbi | AF527608 |
| *Salmonella*_phage_22 | KR296686 |
| Enterobacteria_phage_UAB_Phi20 | NC_031019 |
| *Salmonella*_phage_103203_sal4 | KU927495 |
| *Salmonella*_phage_64795_sal4 | KU927498 |
| *Salmonella*_phage_146851_sal4 | KU927492 |
| *Salmonella*_phage_118970_sal4 | NC_030919 |
| *Salmonella*_phage_146851_sal5 | KU927491 |
| *Salmonella*_phage_103203_sal5 | NC_031946 |
| *Salmonella*_phage_101962B_sal5 | KU927496 |
| *Salmonella*_phage_SJ46 | NC_031129 |
| *Salmonella*_phage_HK620 | NC_002730 |
| *Salmonella*_phage_epsilon15 | NC_004775 |
| *Salmonella*_phage_epsilon34 | NC_011976 |
| *Salmonella*_phage_37 | NC_029045 |
| *Salmonella*_phage_35 | KR296689 |
| *Salmonella*_phage_FSL_SP-039 | KC139514 |
| *Salmonella*_phage_FSLSP030 | NC_021779 |
| *Salmonella*_phage_Chi | NC_025442 |
| *Salmonella*_phage_118970_sal1 | NC_031930 |
| *Salmonella*_phage_BP12C | NC_031228 |
| *Salmonella*_phage_iEPS5 | KC677662 |
| *Salmonella*_phage_FSLSP088 | NC_021780 |
| *Salmonella*_phage_FSL_SP-124 | KC139515 |
| *Salmonella*_phage_SPN19 | NC_019417 |
| *Salmonella*_phage_FSL_SP-016 | KC139516 |
| *Salmonella*_phage_SPN3UB | NC_019545 |
| Phage_Gifsy2 | NC_010393 |
| *Salmonella*_phage_SEN34 | NC_028699 |
| *Salmonella*_phage_NR01 | NC_031042 |
| *Salmonella*_phage_Shivani | NC_028754 |
| *Salmonella*_phage_118970_sal2 | KU927493 |
| *Salmonella*_phage_100268_sal2 | KU927497 |
| *Salmonella*_virus_Stitch | NC_027297 |
| *Salmonella*_phage_7-11 | NC_015938 |
| *Salmonella*_phage_19 | NC_029072 |
| *Salmonella*_phage_41 | KR296695 |
| *Salmonella*_phage_18-India | KR091942 |
| *Salmonella*_phage_phSE-2 | NC_031026 |
| *Salmonella*_phage_phSE-5 | KX015771 |
| *Salmonella*_phage_36 | NC_029071 |
| *Salmonella*_phage_FSL_SP-126 | KC139513 |
| *Salmonella*_phage_FelixO1 | NC_005282 |
| *Salmonella*_phage_FO1a | JF461087 |
| *Salmonella*_phage_Mushroom | KP143762 |
| Enterobacteriaphage_UAB_Phi87 | JN225449 |
| *Salmonella*_phage_HB-2014 | NC_027329 |
| *Salmonella*_phage_BPS15Q2 | NC_031939 |
| *Salmonella*_phage_39 | KR296693 |
| *Salmonella*_phage_SPC32N | KC911857 |
| *Salmonella*_phage_SPN9TCW | JQ691610 |
| *Salmonella*_phage_SPC32H | KC911856 |
| *Salmonella*_phage_SPN1S | NC_016761 |
| *Salmonella*_phage_SPFM16 | LR535916 |
| *Salmonella*_phage_SPFM2 | LR535902 |
| *Salmonella*_phage_SPFM13 | LR535913 |
| *Salmonella*_phage_SPFM15 | LR535915 |
| *Salmonella*_phage_SPFM17 | LR535917 |
| *Salmonella*_phage_SPFM5 | LR535905 |
| *Salmonella*_phage_SPFM21 | LR535920 |
| *Salmonella*_phage_SPFM3 | LR535903 |
| *Salmonella*_phage_SPFM22 | LR535921 |
| *Salmonella*_phage_SPFM14 | LR535914 |
| *Salmonella*_phage_SPFM12 | LR535912 |
| *Salmonella*_phage_SPFM11 | LR535911 |
| *Salmonella*_phage_SPFM9 | LR535909 |
| *Salmonella*_phage_SPFM10 | LR535910 |
| *Salmonella*_phage_SPFM19 | LR535918 |
| *Salmonella*_phage_SPFM8 | LR535908 |
| *Salmonella*_phage_SPFM4 | LR535904 |
| *Salmonella*_phage_SPFM7 | LR535907 |
| *Salmonella*_phage_SPFM6 | LR535906 |
| *Salmonella*_phage_SPFM20 | LR535919 |
| Enterobacteria_phage_14L4 | LC465543 |
| *Salmonella*_phage_SPFM1 | LR535901 |
| *Salmonella*_phage_SPN3US | NC_027402 |
| Enterobacteria_phage_SEGD1 | KU726251 |
| *Salmonella*_phage_phiSG-JL2 | NC_010807 |
| *Salmonella*_phage_BP12A | NC_031258 |
| *Salmonella*_phage_Vi06 | NC_015271 |
| Enterobacteria_phage_vB_KleM-RaK2 | NC_019526 |
| *Salmonella*_phage_BP63 | NC_031250 |
| *Salmonella*_phage_vB_SenMS16 | NC_020416 |
| *Salmonella*_phage_STP4-a | NC_026607 |
| *Salmonella*_phage_STML-198 | NC_027344 |
| *Salmonella*_phage_vB_SnwM_CGG4-1 | NC_031065 |
| *Salmonella*_phage_SSU5 | NC_018843 |
| *Salmonella*_phage_IME207 | NC_031924 |
| Enterobacteria_phage_K1-5 | NC_008152 |
