# Supplementary figures and images for: Genomic Characterization of Jumbo Salmonella Phages That Effectively Target United Kingdom Pig-Associated Salmonella Serotypes
Source: Front Microbiol. 2019 Jul 2;10:1491. doi: 10.3389/fmicb.2019.01491 (PMC6614189; doi:10.3389/fmicb.2019.01491)

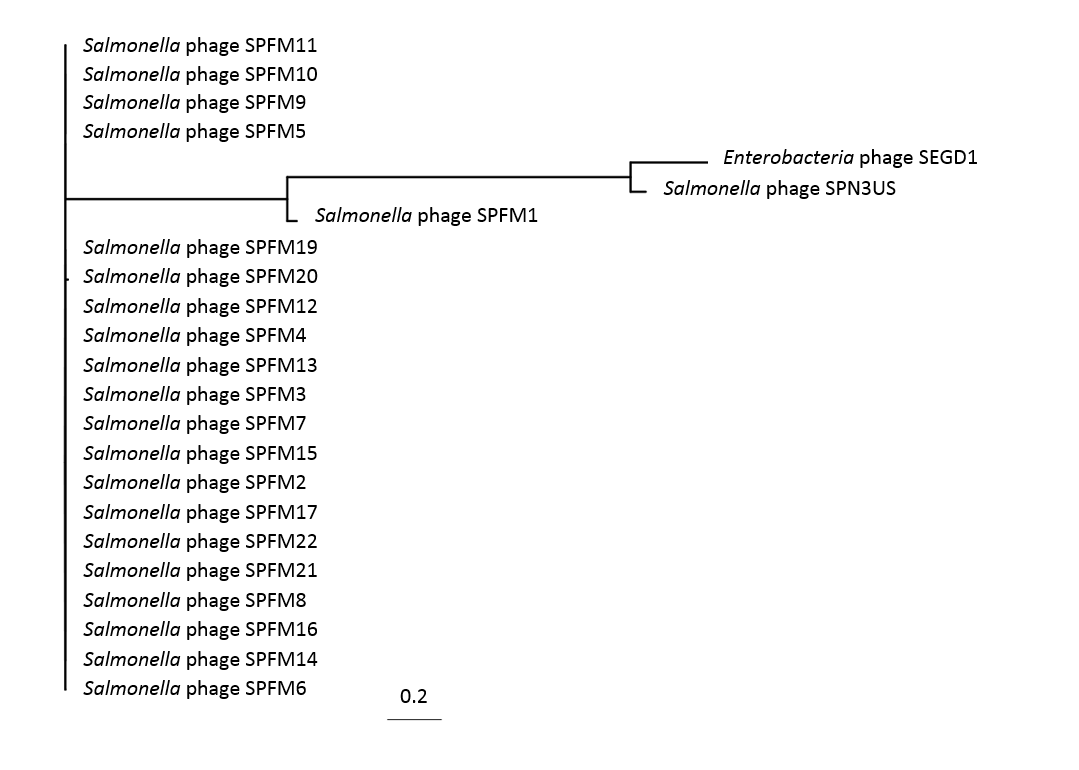

Supplement: Supplementary file 5 [file Image_1.tif]
